# Repurposing Cannabidiol as a Potential Drug Candidate for Anti-Tumor Therapies

**DOI:** 10.3390/biom11040582

**Published:** 2021-04-15

**Authors:** Fei Wang, Gabriele Multhoff

**Affiliations:** 1 Radiation-Immuno Oncology Group, TranslaTUM—Central Institute for Translational Cancer Research, Klinikum rechts der Isar, TU München, Einsteinstr. 25, 81675 Munich, Germany; gabriele.multhoff@tum.de; 2 Department of Oncology, The Second Affiliated Hospital of Zunyi Medical University, Zunyi 563000, China; 3 Department of Radiation Oncology, Klinikum rechts der Isar, TU München, 81675 Munich, Germany

**Keywords:** cannabinoids, cannabidiol, anti-tumor therapy

## Abstract

In recent years, evidence has accumulated that cannabinoids—especially the non-psychoactive compound, cannabidiol (CBD)—possess promising medical and pharmacological activities that might qualify them as potential anti-tumor drugs. This review is based on multiple studies summarizing different mechanisms for how CBD can target tumor cells including cannabinoid receptors or other constituents of the endocannabinoid system, and their complex activation of biological systems that results in the inhibition of tumor growth. CBD also participates in anti-inflammatory activities which are related to tumor progression, as demonstrated in preclinical models. Although the numbers of clinical trials and tested tumor entities are limited, there is clear evidence that CBD has anti-tumor efficacy and is well tolerated in human cancer patients. In summary, it appears that CBD has potential as a neoadjuvant and/or adjuvant drug in therapy for cancer.

## 1. Introduction

In the past few decades, cannabinoids—that can be classified into endogenous cannabinoids, synthetic cannabinoids and phytocannabinoids, derived from the plant *Cannabis sativa* L.—have attracted great interest in medicine with respect to their broad medical applicability [1]. *Cannabis sativa* L. has been used for the treatment of glaucoma, anxiety, nausea, depression and neuralgia [2,3,4,5]. The therapeutic value of phytocannabinoids has been demonstrated in the management of HIV/AIDS symptoms and multiple sclerosis [6,7]. Terpenoid compounds are found in the bulbous glands, capitate-sessile glands and capitate-stalked glands of pistillate flowers [8]. The capitate-stalked type glands contain the highest amount of non-psychoactive cannabinoids, whereas the well-known psychoactive compound tetrahydrocannabinolic acid (THCA) is predominantly found in the glandular cells of the plant.

**Figure 1:**
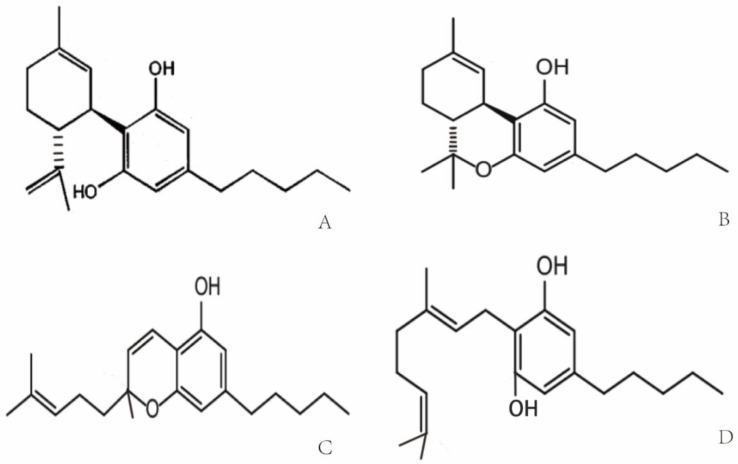
(**A**) Molecular structures of cannabidiol (CBD) [26] (**B**) Δ9-tetrahydrocannabinol (Δ9-THC), (**C**) cannabichromene (CBC), and (**D**) cannabigerol (CBG) [16].

Cannabis was used for the first time for medical purposes in 16th century in Asia for analgesic, anticonvulsant, hypnotic, anti-inflammatory, antitussive and expectorant treatments [9]. In 1840, William O’Shaughnessy noted the medical properties of Indian cannabis for the treatment of different diseases including asthma, insomnia and opium-use withdrawal [10]. Due to an association of the psychoactive potential of cannabis with crime and mental deterioration, the drug was prohibited at the end of the 19th century and other non-psychoactive synthetic derivatives of cannabis have since been produced [11,12]. More than 560 different compounds have been identified in cannabis, each with different biological and chemical activities that might qualify them as potential drug candidates [13]. Phytocannabinoids that interact with the endocannabinoid system have been analyzed extensively [14]. Phytocannabinoids contain high amounts of non-psychoactive compounds such as cannabichromene (CBC), cannabigerol (CBG) and cannabidiol (CBD) [8,15], and lower amounts of psychoactive cannabinoids [16] such as Δ9-tetrahydrocannabinol (Δ9-THC) [8,17,18]. The chemical structures of the compounds are shown in Figure 1. Δ9-THC and CBD are the two best-studied active components of *Cannabis sativa* L., which can either be directly extracted from the plant or synthesized chemically for medical applications [17,19]. Clinical and preclinical trials have shown that CBD [20,21], unlike Δ9-THC, does not induce hallucinogenic effects [22]. Presently, maceration (ME), ultrasound-assisted extraction (UAE) and reflux heat extraction (RHE) are used to isolate phytocannabinoids such as CBD. UAE is superior to other methods with respect to time, energy consumption and costs [23]. Due to its suitable polarity, ethanol (96%) works best as the extraction solvent [24,25].

## 2. Molecular Targets of Cannabidiol (CBD)

CBD, one of the phytocannabinoids of *Cannabis sativa* L., was first identified by Raphael Mechoulam in the 1960s [27]. CBD with a molecular weight of 314.464 g/mol consists of a cyclohexene ring, a phenolic ring and a pentyl sidechain. In addition, methyl, n-propyl, n-butyl, and n-pentyl sidechain homologs are present in CBD [28,29]. The metabolism of CBD starts with a hydroxylation on position C-7, which results in (−)-7-hydroxy-CBD, followed by (−)-7-carboxy-CBD after a further oxidation step [30].

Cannabinoid receptors and other constituents of the endocannabinoid system have also been identified and characterized. Cannabinoid receptor interaction influences different physiological processes such as appetite, pain and inflammation [31,32]. Based on their molecular structure, cannabinoid receptors belong to the guanine nucleotide-binding protein (G protein)-coupled receptor superfamily [33] that stimulates guanosine 5′-*O*-(3-[^35^S]thio)-triphosphate ([^35^S]GTPγS) and thereby decreases the affinity of GDP binding [34,35]. The cannabinoid receptor CB1 contains seven-transmembrane domains, which initiate the mitogen-activated protein kinase via G proteins for cell signaling. The cannabinoid receptor CB2 shares a sequence homology of 48% with CB1. Cannabinoid-specific receptors are expressed on a large variety of different mammalian tissues. In contrast to CB1, CB2 is not expressed on cells of the central nervous system, but on some peripheral neuronal tissues. CB1 and CB2 are both expressed on many primary immune cells [36,37]. Δ9-THC specifically binds to both cannabinoid receptors, CB1 and CB2. In the CB1 receptor-containing endocannabinoid system, Δ9-THC acts as an agonist, but its agonistic potency is significantly lower than that of other cannabinoids receptor agonists (e.g., CP55940 or WIN55212) with a high intrinsic activity [35,38]. The binding of Δ9-THC to the CB2 receptor is weaker than that to the CB1 receptor [39]. Unlike Δ9-THC, CBD acts as a cannabinoid receptor antagonist with low affinity to both receptors [26,40,41], whereas the isomers (+)-CBD exhibit a high affinity to CB1 and CB2 [26]. A study has shown that CBD antagonizes both cannabinoid receptors in the whole-brain membrane tissues of mice, as well as Chinese hamsters’ ovary cells which were transfected with the human CB2 receptor [42]. A few reports have now shown that CBD might act as a negative allosteric modulator of the CB1 and CB2 receptors [43,44] (Table 1). 

Transient receptor potential vanilloid (TRPV) channels are members of the TRP superfamily that modulates the transmission of iron and calcium in cells and thereby mediates a variety of neuronal signaling processes, such as the sensing of temperature, pressure, pH, smell, etc. The occurrence of some common diseases—inflammatory and chronic pain, for instance—are associated with dysfunctional TRPV1 and TRPV2 receptors [26,45]. It is well accepted that CBD has a weak affinity to CB1 and CB2 receptors, but predominantly interacts with TRPV1 and TRPV2 receptors and the mustard oil receptor [26,45,46,47], which acts as a negative allosteric modulator of the CB1 receptor [43,48]. TRPV1 interacts with phosphatidylinositol-3-kinase (PI3K) signaling [49] and thereby supports cell survival. In contrast to TRPV1, TRPV2 is predominantly activated by different phytocannabinoids [50] including CBD [51]. The efficacy of CBD to activate TRPV3/4 is weaker compared to that of TRPV1/2 (~54, ~15 vs. ~78, ~67%, respectively) [52] (Table 1). CBD displays a high potency to agonistically support the TRP ankyrin type-1 (TRPA1) channel’s activity (efficacy ~108%) [52], whereas it antagonizes the TRP melastatin type-8 (TRPM8) channel’s activity [53]. Other molecular targets of CBD that are mediated through the CB1/CB2 receptors include the fatty acid neurotransmitter [54,55,56], G-protein coupled receptors (GPR55/ GPR18) [57,58,59,60], serotonin receptor 5-HT1 [61], peroxisome proliferator-activated receptor γ (PPARγ) [62,63], cyclooxygenase-2 (COX-2) enzymes and glycine-receptor [64]. Furthermore, CBD mediates neuroprotection by influencing cytosolic Ca^2+^ and K^+^ homeostasis by blocking low-voltage-activated (T-type) Ca^2+^ channels [65,66], reducing Ca^2+^ levels, preventing Ca^2+^ oscillations under high-excitability and by altering K^+^ levels [45].

## 3. CBD and Cancer

**Figure 2:**
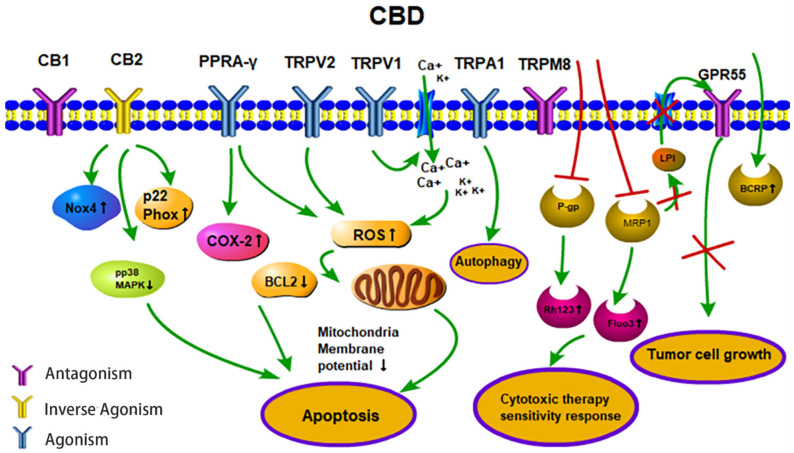
Anti-tumor activities (apoptosis, therapy sensitivity, autophagy, tumor cell growth) of CBD. CBD acts as an agonist for the receptors TRPV1/2, TRPA1 and PPARγ. CBD acts as an inverse agonist of the receptors CB1/CB2 and as an antagonist of the receptors GPR55 and TRPM8. CBD inhibits the efflux transporters P-gp and MRP1 and thereby reverses multi-drug resistance. CBD inhibits the MRP1 pump LPI out and the autocrine loop with GPR55 thereby reduces cell proliferation. Abbreviations: BCRP, breast cancer resistance protein; BCL2, B-cell lymphoma 2; CB1/2, cannabinoid receptors type 1/2; CBD, cannabidiol; COX-2, cyclooxygenase 2; Fluo3, substrate of MRP1; GPR55, G-protein-coupled receptor 55; LPI, lysophospholipid lysophosphatidylinositol; MRP1, multidrug resistance-related protein 1; NOX4, NADPH oxidase 4; PPARγ, peroxisome proliferator-activated receptor-gamma; P-gp, P-glycoprotein; p22 Phox, human neutrophil cytochrome b light chain; Rh123, P-gp substrate rhodamine 123; ROS, reactive oxygen species; TRPV1/2, transient receptor potential vanilloid type 1/2; TRPA1, TRP ankyrin type-1; TRPM8, TRP melastatin type-8.

Cancer has become the second greatest cause of disease-related death worldwide in the past few decades [68]. The major treatment strategies for cancer are based on surgery, chemotherapy and/or radiotherapy. However, normal tissue toxicity and the therapy resistance of tumor cells often limit the efficacy of these three major treatment pillars. Therefore, there is a great medical need for anti-cancer drugs that are well tolerated and highly effective against cancer cells. CBD could serve as a potential anti-cancer drug candidate that fulfills these criteria. In vitro studies with fetal rat telencephalon cells have revealed that highly lipophilic cannabinoids can easily cross the blood-brain barrier and thereby access tumor target cells within the nervous system [69]. Since CB2 receptors mediate neuronal cell survival and proliferation, treatment with the antagonist, CBD, could inhibit the formation and propagation of malignant glial tumor cells [37]. Lipophilic CBD influences mitochondrial calcium stores, glycine receptors and fatty amide hydrolases [70]. Due to its non-psychoactive characteristics, lipophilic CBD has the potential to become an important drug in the treatment of glioblastoma [71]. Preclinical studies have demonstrated that CBD impedes tumor cell proliferation and metastatic spread and induces autophagy and/or apoptosis in tumor cells [62,72]. CBD induces tumor cell apoptosis by activating the pro-caspases-3/8/9 and increases the production of reactive oxygen species (ROS) in human glioma cells. In addition, CBD disturbs redox homeostasis by depleting intracellular glutathione stores via stimulation of the activity of both glutathione reductase and glutathione peroxidase. Interestingly, CBD does not influence the viability of normal primary glial cells [73]. Incubation of the human glioblastoma cell lines, U87 and U373, with CBD reduces their proliferative capacity in a concentration-dependent manner [74]. The partial activation of CB2 receptor activity by CBD is not impaired by the antagonistic activity of the CB1 receptor, and in the absence of the ROS scavenger alpha-tocopherol/vitamin E, the induction of apoptosis by CBD is not inhibited [73,74]. The treatment of leukemia cells with CBD has resulted in CB2 receptor-mediated cell death. CBD treatment results in a disruption of mitochondrial membrane potential, which is accompanied by a release of the pro-apoptotic factor cytochrome c [75]. Other studies have demonstrated that CBD affects the regulation of CB2 and the NAD(P)H oxidases Nox4 and p22 (Phox) [76]. In non-small cell lung cancer (NSCLC) cell lines (A549, H460, H358), the inhibition of the invasive capacity of the tumor cells by CBD has been related to a reduction in plasminogen activator inhibitor-1 (PAI-1) [77]. COX-2 inhibitor (NS-298) and PPAR-γ antagonist (GW9662) studies in lung cancer cell lines (A549, H460) and primary lung cancer cells have demonstrated that CBD-induced apoptosis is associated with an upregulation of the pro-apoptotic markers, COX-2 and PPAR-γ [78]. Activation of the cannabinoid receptors by CBD induces apoptotic cell death in epidermal tumor cells in vitro and results in significant growth inhibition in an epidermal tumor mouse model. Interestingly, the viability of non-transformed normal epidermal cells remains unaffected by CBD [79] (Figure 2). 

Depending on the applied concentration, CBD mediates different pharmacological effects. Low CBD concentrations, in the range of 0.01 µM to 9  μM, do not impair tumor cell viability but result in an increased migratory capacity of U87 glioblastoma cells. In this low concentration range, the biological activities of CBD are neither related to the CB1/CB2 receptors nor the TRPV1 receptor [80], whereas the effects of higher CBD concentrations clearly depend on these receptors [26]. Furthermore, CBD acts as a TRPV2-selective activator by intensifying Ca^2+^ influx and thereby inducing apoptosis in U87 glioblastoma cells [81]. Other studies suggest that CBD may promote doxorubicin-mediated cell death in hepatocellular carcinoma (BNL1 ME) and triple-negative breast cancer (TNBC) cells by facilitating the uptake of doxorubicin, via the activation of the TRPV2 channels [82,83]. Overexpression of TRPV3 in human lung cancer cells correlates with a poor overall survival of lung cancer patients, but blocking TRPV3 efficiently inhibits the proliferation of lung cancer cells [84]. The low binding capacity of CBD to TRPV3 might be another explanation for its anti-tumoral effects. Treatment of the triple-negative breast carcinoma cell line, MDA-MB-231, with CBD induces both, autophagy and apoptosis, as demonstrated by a ROS accumulation, Beclin1 activation and Bcl-2 inhibition [85]. TRPA1/TRPM8 receptors that have been shown to promote autophagy and the metabolic transformation of UCP2 (uncoupling protein 2), respectively, in Lewis lung cancer (LLC) cells [85] might be responsible for CBD-induced autophagy and modulated ROS levels (Figure 2). 

In combination with temozolomide (TMZ, the benchmark drug for the management of GBM), cannabinoids enhance autophagy in U87MG glioblastoma cells in vitro. In xenograft glioblastoma mouse models, a subsequent reduction in tumor size is associated with an increase in active cleaved caspases [86]. Treatment of U251 and SF126 glioblastoma cell lines with both Δ9-THC plus CBD is superior to monotherapy with Δ9-THC with respect to ERK signaling, G0-G1 arrest and repression of tumor cell survival [87]. Additionally, combined treatment has resulted in modulation of the oxidative stress response and the lipoxygenase pathway [80,86,87]. Ligresti et al. have reported an accumulation of intracellular Ca^2+^ and reactive oxygen species (ROS) upon CBD treatment, followed by an induction of apoptosis in MDA-MB-231 cells through either direct or indirect activation of CB2 and/or TRPV1 receptors [88]. Another study in MDA-MB-231 cells shows the induction of autophagy and apoptosis and an increase in the ER stress response, AKT/mTOR pathway and cell cycle arrest by CBD [85]. Moreover, CBD significantly inhibits EGFR/AKT and MAPK/ERK, as well as NF-kB signaling that mediates proliferation and chemotaxis [89]. CBD also inhibits some efflux transporters, such as P-glycoprotein (P-gp) and multidrug resistance-related protein 1 (MRP1) by reversing multiple drug resistance (MDR) in anti-cancer therapies. Decreased P-gp expression correlates with an accumulation of the P-gp substrate Rhodamine 123 (Rh123) and promotes the sensitivity of the human T lymphoblastoid leukemia cell line (CEM/VLB(100)) and a mouse fibroblast MDR1 transfected cell line (77.1) towards the P-gp substrate, vinblastine [90]. Moreover, CBD increases the uptake of intracellular MRP1 substrates, Fluo3 and vincristine in ovarian carcinoma cells [91]. Another study has shown that P-gp efflux function is down-regulated after long-term (72 h) CBD exposure, while the breast cancer resistance protein (BCRP), one of the efflux transporters, is up-regulated in a choriocarcinoma cell line [92]. The MRP1 (ATP-binding cassette transporter) pump LPI (lysophospholipid lysophosphatidylinositol) initializes cascades downstream of GPR55 to induce proliferation and migration in prostate and ovarian cancer cells [93]. CBD has also been shown to inhibit GPR55-mediated migratory and proliferative capacity in breast cancer cells [94]. CBD strongly inhibits the anti-invasive capacity of tumor cells by reducing Id-1 (an inhibitor of basic helix-loop-helix transcription factors), as shown by in vitro and in vivo experiments [95,96]. The intercellular adhesion molecule-1 (ICAM-1) and tissue inhibitor of matrix metalloproteinases-1 (TIMP-1), which are frequently decreased in tumor cells upon treatment, are up-regulated by CBD, whereas an opposite trend is detected for matrix metalloproteinases (MMPs) in lung cancer cells, which obstruct tumor cells’ ability to pass through complex extracellular matrices [97] (Table 2). 

Due to their small size, targeted nanoparticles loaded with drugs show an advantageous biodistribution and dissemination in the body, and have the capacity to release the drug in close proximity to the tumor [98,99]. Although CBD is a potentially effective anti-cancer drug, poor water solubility and the requirement for organic solvents such as ethanol or methanol limit its broader application [100,101]. Due to these limitations, nanoparticles loaded with CBD (CBD-NPs) at a ratio of 1:5 or 3% (*w*/*w*) have first been tested to treat ovarian cancer cells in vitro and for in vivo models. Compared to free CBD in organic solvents, CBD-NPs induce apoptosis and tumor growth inhibition at lower IC50 values [102]. CBD in lipid nanocarriers exhibits a high brain targeting ability by enhancing the passage across the blood-brain barrier in vitro and in ovo and, therefore, might provide a novel strategy to treat CNS tumors [103].

**Table 2:** Studies summarizing the multiple effects of CBD.

Type/Cancer	Cell Line	Mechanism	Conclusion	Ref.
glioblastoma	U87/U373 in vitro/in vivo	ssDNA↑	proliferation↓ apoptosis	[74]
prostate ovarian	PC-3/DU145 OVCAR3	GPR55/LPI↓ phospho ERK1/2↓	proliferation↓ cell migration↓	[93]
glioblastoma	U87	ROS↑ caspase↑ cytochrome c↑ CB1/CB2/TRPV1 receptor-independent TRPV2-activation Ca^2+^ influx↑	apoptosis↑ migration↓ viability↓ (no effect at low doses)	[73,80,81]
glioblastoma	U251/SF126 in vitro/in vivo	ERK↓ G0-G1 arrest ROS↑ Id-1↓	cell survival↓ proliferation↓ apoptosis↑ aggressiveness↓	[87,95]
acute T lymphoblastic leukemia	Jurkat/MOLT-3/CCFR-CEM/K562/Reh/RS4	mito Ca^2+^ overload mito transition pore formation↑ ROS↑ cytochrome c↑	autophagy↑ apoptosis↑	[75]
breast	MDA-MB-231/MCF-7	AKT/mTOR↓ BCL2↓ ROS↑ beclin1↑ direct/indirect activation CB2 and/or TRPV1 intracellular Ca^2+^↑ AKT/mTOR↓ cell cycle arrest GPR55↓ LPI↓	apoptosis/ autophagy↑ migration↓	[85,94]
breast	SUM159/4T1/SCP2/MVT-1/MDA-MB-231/RAW 264.7	GM-CSF/CCL3↓ NF-κB↓ EGFR/AKT↓ MAPK/ERK↓	cell growth↓ metastasis↓ TME	[89]
lung	A549/H460/H358 in vitro/in vivo	PAI-1↓ COX-2/PPAR-γ↑ ICAM-1↑ TIMP-1↑ MMP↓	invasiveness↓ apoptosis↑	[77,78,97]
T lymphoblastoid leukemia	CEM/VLB(100)	P-gp↓ Rh123↑	sensitivity↑	[90]
ovarian	2008	MRP1↓ Fluo3/vincristine↑ GPR55↓ LPI↓	sensitivity↑ proliferation↓	[91,93,104]
immune cells	T/macrophages/NK cells	IFN-γ↓ IL-2↓ TNF-α↓ IL-10↑ GM-CSF↓ CCL3↓ NFAT↓	proliferation↓ infiltration↓	[89,105,106,107,108]
primary endothelial cells	HUVEC	VEGF-2/VEGFA-2↓ MMP-2/9↓ uPA↓ ET-1↓ PDGF-AA↓ CXCL16↓ ERK/Akt↓ JNK↓	angiogenesis↓	[109,110]

Abbreviations: BCRP, breast cancer resistance protein; BCL2, B-cell lymphoma 2; CB1/2, cannabinoid receptors type 1/2; CCL, chemokine (C-C motif) ligand 3; COX-2, cyclooxygenase 2; CXCL16, chemokine ligand 16; EGFR/AKT, epidermal growth factor receptor signaling pathway; ET-1, endothelin-1; Fluo3, substrate of MRP1; GPR55, G-protein-coupled receptor 55; GM-CSF, granulocyte-macrophage colony-stimulating factor; IFN-γ, interferon-γ; IL, interleukin; Id1, inhibitor of differentiation/DNA binding; ICAM-1, intercellular adhesion molecule 1; LPI, lysophospholipid lysophosphatidylinositol; mito, mitochondrial; MAPK, mitogen-activated protein kinase pathway; MMP, matrix metalloproteinase; MRP, multidrug resistance-related protein; NF-κB, nuclear factor kappa-light-chain-enhancer of activated B cells; NK, natural killer; NFAT, nuclear factor of activated T-cells; PAI-1, plasminogen activator inhibitor type 1; PDGF-AA, platelet-derived growth factor-AA; P-gp, P-glycoprotein; PPAR-γ, peroxisome proliferators activated receptor gamma; Rh123, rhodamine 123; ROS, reactive oxygen species; ssDNA, single stranded DNA; TNF, tumor necrosis factor; TIMP-1, metallopeptidase inhibitor 1; TRPV1/2, transient receptor potential vanilloid type 1/2; uPA, urokinase-type plasminogen activator; VEGF-2/VEGFA-2, vascular endothelial growth factor 2 and angiopoietin 2.

## 4. Tumor Microenvironment

The immune system plays a critical role in controlling tumor cells. This immunosurveillance largely depends on the synergy of cytotoxic T cells (CTLs) and natural killer (NK) cells, as well as dendritic cells (DCs) and helper CD4+ Th1 cells [111]. However, a large variety of “tumor immune escape” mechanisms often hinder long-term protective anti-tumor immunity [112]. Over-stimulated, pro-inflammatory immune cells can induce a chronically inflamed tumor microenvironment, which supports tumor progression [113], such as ulcerative colitis inducing colorectal cancer [114] and chronic bronchial inflammation-inducing lung cancer [115]. Studies indicate that complex extracellular and intracellular signaling pathways, including the epidermal growth factor (EGF)/mTOR, NF-κB/STAT3 and JNK pathways, are activated during tumor cell transformation, as promoted by chronic inflammation [116,117,118]. CBD suppresses the activation of the EGF/EGFR signaling pathway and its downstream targets Akt, ERK and NF-κB in multiple tumor cells, which demonstrates that there might be a potential role for CBD to interfere in the cross-talk between inflammation, cell survival and tumor proliferation [85,89,119]. Preclinical and first clinical trials combining chemotherapy and anti-inflammatory drugs such as cyclooxygenase-2 (COX-2) inhibitors are already used to prevent tumor progression in prostate or breast cancer [120]. In addition, in xenograft glioma tumor mouse models, CBD induces tumor cell death by modulating the LOX/COX-2 pathway [121].

It has been shown CBD not only induces cell death in cancer cells but also alters the reactivity of some immune cells. CBD suppresses the proliferation and infiltration of T cells and macrophages in the tumor microenvironment, reduces the production of pro-inflammatory cytokines—such as interferon-γ (IFN-γ), interleukin-2 (IL-2) and tumor necrosis factor-α (TNF-α)—and increases that of immune-suppressive cytokines, such as interleukin-10 (IL-10), in vitro and for in vivo models [105]. This hints that CBD inhibits immune effector cell functioning [106]. Another study demonstrated that the CBD-induced cell death was mediated via the inhibition of voltage-dependent anion channel 1, located in the mitochondrial membranes of immortalized BV-2 microglial cells [122]. In triple-negative breast cancer cells, CBD causes the down-regulated release of GM-CSF and CCL3 cytokines, which are important for macrophage recruitment and activation [89] (Table 2).

CBD also has the capacity to down-regulate the release of anti-inflammatory or pro-inflammatory mediators [104]. Due to the agonistic activity of CBD on serotonin 5-HT1 receptors, TRPV1 and the glycine signaling pathway [50,61,123], the generation of ROS and the release of pro-inflammatory chemokines and cytokines can be inhibited, along with T cell proliferation [124,125]. Repeated injections of CBD (1.5 mg/kg IP) into animals for 10 weeks lead to reduced T cell differentiation and invasion, which is accompanied by a decrease in the release of pro-inflammatory cytokines and chemokines [126]. Another study has shown that CBD causes the suppression of NFAT, in addition to common pro-inflammatory cytokines such as IL-2 and IFN-γ, suggesting an inhibition of the activity of NK cells and/or T cells [106,107,108,127] (Table 2). GPR55, another target of CBD, exhibits anti-inflammatory activities that depend on intracellular pathways such as RhoA-dependent Ca^2+^ signaling, ERK/NF-kB and the p38 MAPK pathways [127,128,129].

## 5. Cannabinoids Inhibit Angiogenesis

Tumor blood vessel neoangiogenesis plays an essential role in the process of tumor formation, expansion and growth. Blood vessels are important for the transport of nutrients and oxygen and also provide appropriate conditions for tumor invasion and distant metastasis. Anti-angiogenic therapies have been extensively tested to suppress tumor growth. CBD effectively inhibits the growth of different types of tumors in vitro and in vivo by down-regulation of vascular density via interference with pro-angiogenic pathways in many solid tumor cells [130,131,132]. Due to its favorable pharmacological and toxicological profile, CBD might provide a new strategy for the design of a cannabinoid-based anti-angiogenic tumor therapy that targets the tumor microvasculature. This process can be modulated by targeting several key factors, such as by inhibiting growth factors like vascular endothelial growth factor (VEGF), integrins or angiopoietins, or by activating inhibitory effector molecules such as thrombospondin or interferons [110]. The anti-angiogenic property of CBD has been confirmed in HUVEC cells by inhibiting pro-angiogenic factors, vascular endothelial growth factor and angiopoietin-2 (VEGF-2/VEGFA-2), matrix metalloproteinase-2/9 (MMP-2/9), urokinase-type plasminogen activator (uPA), endothelin-1 (ET-1), platelet-derived growth factor-AA (PDGF-AA) and chemokine ligand 16 (CXCL16), as determined by immunohistochemical analyses and vascular permeability assays [109,133]. Cannabinoid anandamide inhibits basic fibroblast growth factor (bFGF)-stimulated endothelial cell proliferation in a dose-dependent manner and induces vessel apoptosis [134]. Some critical pro-survival markers and migratory pathways such as ERK/Akt phosphorylation, FAK, JNK signaling and the activity of matrix metalloprotease MMP-2 result in impaired endothelial cell proliferation, migration and tube formation [110]. Moreover, the p38/MAPK signaling pathway is also involved in CBD-induced apoptosis [110,134]. Other researchers report reduced sprouting activity of endothelial cells in spheroids, as well as direct induction of apoptosis in endothelial cells by CBD, both in cell cultures and in chick chorioallantoic membrane assays [132,134]. CB1 receptors stimulate corresponding intracellular signaling during angiogenesis [110]. Some studies suggest that even at low nanomolar concentrations, CBD binds to the cannabinoid receptors CB1/CB2, antagonizes receptor agonists, and thereby blocks the anti-angiogenic effect, which is mediated in part by CB1/CB2 [42]. However, further studies are necessary to elucidate the exact molecular mechanism of CBD on the microvasculature.

In non-melanoma skin cancer, which is believed to arise mainly from the stem cells of hair follicles [135], CB1 and CB2 receptors are expressed. EGF-R phosphorylation is markedly reduced upon CBD treatment; large parts of the tumor vessels shrink to narrow capillaries, compared to control carcinomas with large, dilated vessels. However, these effects appear not to be related to apoptosis [79]. Another study suggests that CBD also inhibits HIF-1α, which is extensively up-regulated in nearly all solid tumors due to a hypoxic tumor microenvironment [136].

## 6. Clinical Trials with CBD

Based on preclinical studies, CBD appears to be a potent anti-cancer agent, one which might qualify for clinical applications in combination with other therapies [137]. Due to its non-psychoactive characteristics, CBD is superior to other cannabinoids in clinical applications. A double-blind, placebo-controlled, randomized clinical phase I trial has been started to prove its safety and capability to relieve tumor-related adverse effects at a CBD concentration of 100 mg/mL, within a dose range of 50 mg to 600 mg per day, in 2 weeks [138]. Another study focused on therapy for severe cancer-related side effects associated with chemotherapy, in which 177 patients were included who were experiencing unsatisfactory analgesic effects even during a standard chronic opioid titration phase. A combination of THC:CBD significantly alleviated their pain intensity compared to the placebo group (23 [43%] vs. 12 [21%]) and also showed weak positive effects with respect to the avoidance of avoiding nausea and vomiting, while the effects in the THC-monotherapy group were non-significant [139]. Some other cannabinoids including Δ-9 tetrahydrocannabinol, cannabidiol and a THC-11-oic acid analog have also been tested for their capacity to reduce the neuropathic pain caused by chemotherapy, and have shown favorable analgesic properties [140,141,142,143]. A phase II clinical trial investigated the curative effects of THC:CBD when combined with dose-intensified TMZ, as versus TMZ mono-chemotherapy, in patients with glioblastoma multiforme (GBM) (clinical trial NCT01812603). In this study, a total of 21 patients diagnosed with GBM were included. Patients received 27 mg/mL THC and 25 mg/mL CBD in 100 µL per day. The TMZ monotherapy group reached a one-year survival rate of 44%, while the THC:CBD plus TMZ group presented promising results, with a one-year survival rate of 83% and a significantly extended median survival of more than 22 months (THC:CBD+TMZ) vs. 12.3 months (TMZ) [71]. In another remarkable study recruiting 119 patients with prostate, breast, esophageal and brain cancer and lymphoma, patients received an intermittent administration of CBD—’three days on and three days off’ CBD therapy—with an average dose of 10 mg twice a day for at least 6 months and a follow-up period of four years. The major findings of this study were that the dose administration should be adapted to the tumor growth rate and diagnosis; the maximum dose of 30 mg should be given to patients with a PD (progressive disease), while the minimum maintenance dose of 5 mg should be given to patients with a SD (stable disease) after chemotherapy. This study failed, however, to define the maximum tolerated dose since no side-effects were seen at the maximum applied dose [144].

## 7. Conclusions

This review provides an update on data associated with the in vitro and in vivo effects of CBD in anti-tumor therapies (Figure 2). Pharmacological effects, as well as complex mechanisms of CBD on activated biological up- and/or downstream targets, were studied with respect to their anti-tumor capacities. Recently, a novel inverse molecular docking approach has been successfully established when exploring the anti-cancer effects of the natural compound, curcumin [145,146]. The related technology might help to provide new insights into the molecular modes of action of CBD.

It has been shown that CBD, either alone or in combination with other therapies, has the potential to act as a novel anti-tumor, anti-inflammatory and anti-pain drug in preclinical studies and first clinical trials. A few clinical trials have now demonstrated beneficial pharmacokinetic and pharmacodynamic characteristics of the drug, and some anti-tumor activities at well-tolerated doses. Therefore, it can be assumed that CBD might be considered a potential candidate for neoadjuvant and/or adjuvant interventions in oncology. However, future studies with larger patient cohorts are necessary to prove the efficacy of CBD in tumor patients.

## Figures and Tables

**Table 1:** Affinity of the receptors to CBD.

Receptor	Effect	Affinity	Sequence Identity
TRPV1	agonist	~78% [52]	79% [52]
TRPV2	agonist	~67% [52]	96% [52]
TRPV3	agonist	~54% [52]	77% [52]
TRPV4	agonist	15% [52]	68% [52]
TRPA1	agonist	108% [52]	~30% [52]
TRPM8	antagonist	IC50 = 70–160 nM [53]	~30% with TRPV2 [52]
CB2	inverse agonist	Ki = 4200 nM [53]	N
CB1	antagonist	Ki = 4900 nM [53]	N
GPR55	antagonist	IC50 = 445 nM [67]	N

Sequence identity, percentage of receptor sequence homology within the putative CBD binding site; N, not defined.
