# Correction: Wang, F.; Multhoff, G. Repurposing Cannabidiol as a Potential Drug Candidate for Anti-Tumor Therapies. *Biomolecules* 2021, *11*, 582

**DOI:** 10.3390/biom13020225

**Published:** 2023-01-24

**Authors:** Fei Wang, Gabriele Multhoff

**Affiliations:** 1 Radiation-Immuno Oncology Group, TranslaTUM—Central Institute for Translational Cancer Research, Klinikum rechts der Isar, TU München, Einsteinstr. 25, 81675 Munich, Germany; 2 Department of Oncology, The Second Affiliated Hospital of Zunyi Medical University, Zunyi 563000, China; 3 Department of Radiation Oncology, Klinikum rechts der Isar, TU München, 81675 Munich, Germany

There was a misplaced reference in the original article in the first paragraph of Section 6 “Clinical Trials with CBD” [1]. By misplacing reference 138 in the original article, an inaccurate interpretation of the study was made.

The amendment is listed below, to which in addition, a sentence has been removed in order to maintain the paragraph’s readability:

“Due to its non-psychoactive characteristics, CBD is superior to other cannabinoids in clinical applications. A double-blind, placebo-controlled, randomized clinical phase I trial has been started to prove its safety and capability to relieve tumor-related adverse effects at a CBD concentration of 100 mg/mL, within a dose range of 50 mg to 600 mg per day, in 2 weeks [138].”

The authors apologize for any inconvenience caused and state that the conclusions of the original article have been unaffected. The original publication has been updated. 
